# Development and psychometric properties of health care workers’ concerns in infectious outbreaks scale

**DOI:** 10.3389/fpsyg.2022.1108835

**Published:** 2023-01-17

**Authors:** Sajad Yarahmadi, Mojgan Khademi, Farzad Ebrahimzadeh, Tayebeh Cheraghian, Elham Shahidi Delshad

**Affiliations:** ^1^Social Determinants of Health Research Center, School of Nursing and Midwifery, Lorestan University of Medical Sciences, Khorramabad, Iran; ^2^Student Research Committee, Semnan University of Medical Sciences, Semnan, Iran; ^3^Nutritional Health Research Center, School of Health and Nutrition, Lorestan University of Medical Sciences, Khorramabad, Iran; ^4^Cardiovascular Research Center, Shahid Rahimi Hospital, Lorestan University of Medical Sciences, Khorramabad, Iran

**Keywords:** concern, exploratory factor analysis, infectious disease outbreaks, psychometry, reliability, validity

## Abstract

**Introduction:**

Healthcare workers are a crucial workforce; from a moral perspective, understanding their concerns and how to support them is crucial and makes it possible for health services to keep functioning. This study aimed to develop and validate Health Care Workers’ Concerns in Infectious Outbreaks Scale (HCWCIOS).

**Methods:**

This exploratory sequential mix-method study was employed to design and validate the HCWCIOS. The initial tool was designed after searching similar studies and performing a qualitative phase under the semi-structured approach. Both qualitative and quantitative methods were used to evaluate the face and content validity. The content validity ratio, content validity index, and item-level content validity index were also calculated. Exploratory factor analysis was employed to evaluate the construct validity. Using a convenient sampling method, 354 Iranian healthcare workers participated in the study. Computing Cronbach’s alpha coefficient estimated the internal consistency for HCWCIOS and its subscales. Furthermore assessed was test–retest reliability.

**Results:**

The preliminary scale was designed with 57 items. By eliminating nine items in the content validity phase and 12 items during factor analysis, the final 36-item scale was developed on six factors: inadequate preparedness, lack of knowledge, risk perception, affected social relations, work pressure, and absenteeism. These six factors accounted for 46.507% of the total variance. The whole scale’s Cronbach’s alpha coefficient was 0.912, and the intra-class correlation coefficient was 0.88.

**Conclusion:**

A 36-item HCWCIOS has good psychometric properties and is suitable for measuring healthcare workers’ concerns during a pandemic.

## 1. Introduction

Increased outbreaks of infectious diseases in recent years, including Severe Acute Respiratory Syndrome (SARS) in 2003, Novel Influenza A/H1N1 in 2009, and Middle East Respiratory Syndrome (MERS) in 2012, have raised concerns about the potential of a global pandemic. The emergence of Corona Virus Disease-2019 (COVID-19) brought this potential to realization (Fernandez et al., 2020) and caused a tremendous public health crisis (Que et al., 2020; Sperling, 2021).

Healthcare workers (HCWs) are on the front lines of the fight against the crisis of such infectious outbreaks (Abolfotouh et al., 2017). They often risk contracting pathogens (Temsah et al., 2020). Therefore, during the past and current infectious outbreaks, frontline HCWs became infected, and many have lost their lives (Chakravorty et al., 2020; Jalili et al., 2021). COVID-19 infections and deaths among HCWs follow that of the general population worldwide, and over 150,000 infections and 1,400 deaths were reported until 2020 (Bandyopadhyay et al., 2020). According to a recent Iranian study, COVID-19 has killed about 10,000 HCWs (Jalili et al., 2021).

Participating in frontline work and receiving such negative information appears to be significant risk factors for psychological distress and problems (Goulia et al., 2010). High levels of concern have been reported in many studies (Alsubaie et al., 2019) in both frontline and non-frontline HCWs (Sahashi et al., 2021). It has significantly impacted them professionally and personally (Moseley et al., 2020). Understanding HCWs’ concerns and how to support them is crucial, not only from a moral perspective but also to ensure that health services remain on track (Borek et al., 2022). The HCWs’ concerns mean facing challenges, fears, and anxieties (Heidarijamebozorgi et al., 2021).

Many frontline HCWs’ are concerned about well-reported deaths, according to media reports in the United Kingdom (Chakravorty et al., 2020). They are frequently concerned about their health and the health of their families, concerned about how they function, and fear being stigmatized (Goulia et al., 2010). A survey of over 10,000 HCWs during the SARS outbreak (2003) reported that many respondents experienced social stigmatization. Nearly half (49%) and 31%, respectively, believed that “people avoid me because of my employment” and “people avoid my family members because of my job.” For instance, some parents of school-aged children prohibited their kids from playing with or being close to HCWs’ kids. A significant portion of HCWs (69%) also thought that “those close to me are concerned they might contract the virus from me” (Koh et al., 2005).

These are only part of the concerns of HCWs during pandemics. Various studies reported that during the outbreak of pandemics, widespread concerns are created among health workers, which becomes a big challenge for health systems in crisis periods (Abolfotouh et al., 2017; Khademipour et al., 2017; Berkhout et al., 2021).

The novelty of the diseases, the lack of prior experience, and the potential that HCWs were not fully informed about the management difficulties by the pertinent authorities during their teaching campaign all could be attributed to a high level of concern (Alsubaie et al., 2019).

Higher job stress, social isolation, and health fears have all been related to HCWs’ concerns and psychological distress around the outbreaks (Goulia et al., 2010; Sheikhbardsiri et al., 2022). Unrecognizing emotions and concerns may prevent patient-centered care, neglect patients’ psychological issues, avoid bonding with patients, and inhibit the quality of care. It also could affect the HCWs’ sense of well-being and may lead to distress, disengagement, job conflict, and burnout (Barello et al., 2020). In an extended crisis such as the pandemic, the sustainability of the healthcare response entirely depends on its capability to protect the health of responders and the HCWs (Muller et al., 2020). However, even when supplied for free or at a low cost, the support uptake by HCWs has remained limited (Berkhout et al., 2021). For an appropriate epidemic response, it is vital to understand the concerns, behaviors, and knowledge of HCWs. The concerns may affect HCWs’ overall effectiveness and must be addressed by including organization policies in outbreak planning (Alsubaie et al., 2019).

Limited studies focus on HCW’S perception of concerns and worries in the past (Wong et al., 2008; Goulia et al., 2010; Abolfotouh et al., 2017) and current pandemics (Koh et al., 2005; Chaudhary et al., 2020; Kinariwala et al., 2020; Sperling, 2021) among different groups of HCWs. In these studies, questionnaires have been used as a data collection tool. However, there is some ambiguity regarding their creditability.

In Singapore, a study evaluated how HCWs perceived the risk and its effects on their work and personal life. A three-part questionnaire has applied, including individual characteristics, 88 questions about the perceived risk of infection, the perceived impact of the SARS pandemic on personal and professional life, and the impact of events scale (Koh et al., 2005). After 3 years of an avian influenza pandemic, Wong et al. (2008) from the same country used a modified version of that questionnaire to study concerns, perceived impact, and preparedness in HCWs (Wong et al., 2008). In MERS outbreaks in Saudi Arabia, the level of concern among HCWs was assessed with Wong’s et al. (2008) instrument (Abolfotouh et al., 2017). Based on retrieved studies, research in Greek is the only article on past outbreaks that report Cronbach’s α score. In this study, Goulia et al. (2010) designed the questionnaire based on the information in the literature about the perspectives and opinions of experts on infectious disease outbreaks (Goulia et al., 2010).

With the emergence of COVID-19, HCWs’ concerns and worries have become the focus of some researchers again. A modified version of Goulia et al.’s (2010) instrument without validity assessment has been used to survey worries and concerns among HCWs in COVID-19 in Japan (Sahashi et al., 2021). Researchers have applied a modified version of Wong’s et al. (2008) scale to study concerns, perceived impact, and preparedness of oral HCWs. In this study, Cronbach’s α score has been reported (Chaudhary et al., 2020). An instrument with no psychometric report was applied to study the concerns and fears of Indian dentists regarding professional practice (Kinariwala et al., 2020).

This brief review of applied questionnaires to study concerns of HCW shows no clear, sufficient, and substantial evidence on the rigorous process of designing and psychometric evaluating of used questionnaires. There is no valid and reliable tool to evaluate HCWs’ concerns in an infectious disease outbreak. More objectively, among those already built instruments, no one validated for the Iranian context can evaluate the healthcare workers’ concerns. Thus, we seek to contribute by filling this gap and offering an instrument to healthcare workers’ concerns in infectious outbreaks, given the relevance and urgency of the matter. Hence, this study aimed to develop and psychometrically evaluate a scale to measure HCWs’ concerns in infectious outbreaks.

## 2. Materials and methods

### 2.1. Study design

This exploratory sequential mix-method study (qualitative-quantitative) was employed to develop and validate the HCWCIOS in Iran during the COVID-19 pandemic in 2020. This study was carried out in two stages: (1) item generation based on literature review and qualitative study findings and (2) psychometric analysis of the developed scale. The data in the first stage were collected by reviewing related literature and performing semi-structured interviews with HCWs.

### 2.2. Item generation

The item generation phase consisted of three steps: (1) a Literature review to find out the concept dimensions; (2) Carrying out a qualitative study to discover other dimensions of HCWs’ concerns in infectious outbreaks that were not fully obtained in the previous step in order to generate the item pool; and (3) Designing the initial tool.

#### 2.2.1. Literature review

In this step, a literature review was applied to identify prior researches that discussed the HCWs’ concerns about infectious outbreaks. The search was conducted in Web of Science, Scopus, and PubMed databases, as well as Iranian ones and Google Scholar search engine, without any time limitation. There were no restrictions regarding the study design. Studies were included if they addressed the concerns of healthcare workers during epidemics and contagious diseases. The statements were extracted to be used as the initial items.

#### 2.2.2. Qualitative phase

Nine HCWs (six female and three male) with strong communication skills and willingness to participate in a study were recruited for the qualitative phase. In this phase, individual interviews under the semi-structured approach were conducted regarding the participants’ preferences. Due to the importance of having different points of view, it was tried to choose participants with maximum diversity in terms of parameters such as gender, job categories, and work experience. Purposive sampling was used to select HCWs. First, the participants received a written consent form, which they must read and sign. The researcher had a pre-prepared interview guide with key questions to better manage the interview time. The interviews were started by asking general questions such as “Tell us about your experiences working during the pandemic?” Through which the participant was allowed to talk openly about the topic. Following the main questions generated from our literature review, exploratory questions were asked. At this point, content analysis was utilized. After transcription, researchers read the written interviews several times to get immersed in the data. They examined the data to identify their preconceptions to build self-reflexivity. To make sense, the researchers frequently asked Wh-questions while performing the analysis. The data analysis and coding were performed. The codes, subcategories, and categories were derived from the transcript data. The researchers also conferred with team members regarding the themes and codes they had retrieved, followed by a thorough explanation of the data analysis procedure and precise citations. The inclusion criteria were: hospital staff who had direct or indirect contact with COVID-19 patients had at least 1 month of work experience during the pandemic and were willing to participate in the study. The interviews continued until the data were saturated because the sample size for qualitative studies could not be determined (Polit and Yang, 2016).

#### 2.2.3. Synthesis stage and designing the initial scale

With the information obtained from the previous two steps and by putting them together (literature review and qualitative interviews), an item pool was created, which was used to build the primary scale in this step.

### 2.3. Psychometric evaluation

In this study stage, face validity and content validity were assessed. Then, the tool’s psychometric properties were then examined in a descriptive cross-sectional study.

#### 2.3.1. Face validity

Face validity is the extent to which a test appears to assess what it is intended to measure (Johnson, 2021). At this stage, the newly designed scale was completed by 10 HCWs. The item impact score was evaluated to determine the quantitative face validity. The item will be retained and considered suitable for further analysis if the impact score exceeds 1.5 (Polit and Yang, 2016). A 5-point Likert scale was used for calculating the item impact score by 10 HCWs that were requested through convenience sampling to study the items. The categories of unimportant (1), slightly important (2), relatively important (3), important (4), and very important (5) were taken into consideration (Cresswell and Plano Clark, 2011). In the qualitative phase of face validity, regarding the items that scored 1.5 or less, the same 10 participants of the quantitative stage were interviewed face-to-face about the items’ difficulty, relevancy, and ambiguity (Ebadi et al., 2017).

#### 2.3.2. Content validity

Both qualitative and quantitative methods were used to evaluate content validity. In the qualitative phase, 20 faculty members with experience in instrument development, patient care, and psychology were requested to evaluate and provide feedback on the items’ wording, item allocation, and scaling. Then, the Content Validity Ratio (CVR) and Content Validity Index (CVI) were computed. Twenty experts were asked to score each item on a three-point scale for reporting CVR as “necessary,” “useful but not necessary,” and “unnecessary.” The items with a CVR of 0.62 and higher were conserved according to Lawshe’s table (Lawshe, 1975). The CVI for each item (I-CVI) and modified kappa coefficient were calculated based on the scoring of the same expert panel. A CVI value of 0.79 or higher was considered optimal without any need to be re-reviewed in the final version. Items with a kappa index less than 0.74 also were deleted. Furthermore, the scale-level CVI (S-CVI) was estimated. If S-CVI/Ave is 0.9 and higher, the scale’s content validity is reported as favorable (Polit et al., 2007).

#### 2.3.3. Construct validity

The final scale was distributed to HCWs to construct validity. Sampling was done by convenience method. The inclusion criteria were: hospital staff directly or indirectly in contact with COVID-19 patients had at least 1 month of work experience during the pandemic and were willing to participate in the study. An incomplete questionnaire was considered as an exclusion criterion. Maximum Likelihood Exploratory Factor Analysis (MLEFA) with varimax rotation was used to assess the scale’s construct validity. The univariate and multivariate normal distributions of data were examined by Skewness (±3) and Kurtosis (±7). Sample adequacy was determined through the Kaiser–Meyer–Olkin (KMO) and Bartlett’s tests. KMO values above 0.7 were considered acceptable (Pahlevan Sharif and Sharif Nia, 2020). The minimum sample size needed for factor analysis is 300, according to Tabachnick et al. (2007). Due to the spread of COVID-19 and the lack of in-person access to the respondents, the questionnaires were provided electronically. From May to November 2020, 304 questionnaires were gathered. The number of factors was calculated using the “Eigenvalue” and “Scree Plot” techniques. Each factor that was extracted from the factor analysis required to be loaded at least 40% to remain constant. More than one eigenvalue was considered (Saggino and Kline, 1996).

#### 2.3.4. Reliability

Cronbach’s alpha (≥0.7) was determined to assess the scale’s internal consistency and that of its subscales (Grove et al., 2012). Fifty HCWs participated in this stage. The test–retest method was used to assess the scale’s stability, and the Intra-Class Correlation (ICC) coefficient >0.8 was an acceptable, two-way mixed model with an absolute agreement in the second round (Polit and Yang, 2016). In this way, the test–retest method was used. A validated scale was given to 30 HCWs, and they were asked to answer the items on a 6-point Likert scale (1 = completely disagree, 6 = completely agree). After 7 days, the same questionnaire was again provided to the HCWs, and they were asked to answer, then the ICC coefficient was calculated. IBM SPSS Amos 25 was used to perform all statistical analyses.

## 3. Results

### 3.1. Item generation

After reviewing related studies, 49 items were obtained by combining and changing the items of similar instruments (Koh et al., 2005; Wong et al., 2008; Abolfotouh et al., 2017). Interviews with nine participants led to the formation of 520 codes, 32 sub-themes, and six themes. Based on the obtained codes, eight items were extracted. The HCWCIOS preliminary item pool was created using the extracted themes, primary categories, and existing literature. The initial scale, 57 items, was then ready for the psychometric procedure.

### 3.2. Psychometric evaluation

Eight items had an impact factor of 1.5 or below, according to the evaluation of face validity. These items were revised in the qualitative stage, and after the reforms, they were returned to the item pool. All the modifications suggested by experts were used in the qualitative review of content validity. The items with a numerical value of less than 0.62 were eliminated following the CVR results. Based on the overall content validity results, nine items were removed, and 48 items reached the item analysis stage. Noteworthily, the S-CVI/Ave scale was obtained as 0.93. At the stage of item analysis, estimates put Cronbach’s alpha at 0.947, and no items were deleted.

Based on the inclusion criteria, 304 HCWs completed the electronic questionnaires. The participants’ average age was 32.25 (SD = 7.34) years. The majority of participants were women (67.10%) with a bachelor’s degree (70.40%) and married (60.20%). The participants had a mean work experience of 10.18 (SD = 6.42) years. We observed that 88.10% of the participants contracted with patients directly, and 55.30% were nurses.

**Figure 1:**
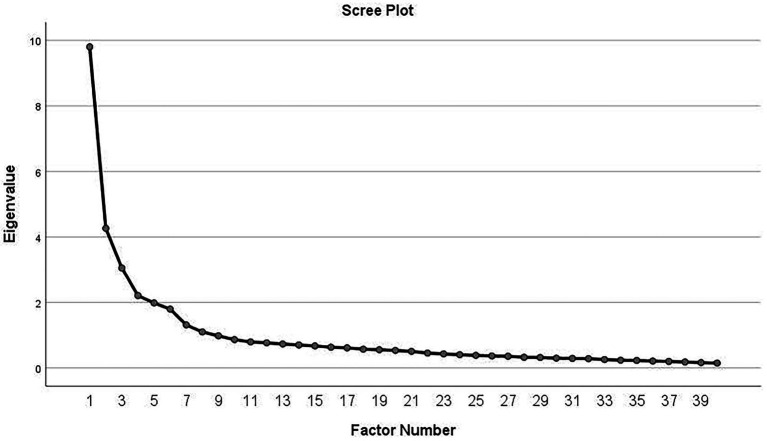
Scree plot. Based on the scree plot, six factors were proposed for extraction in the EFA of the HCWCIOS.

Exploratory Factor Analysis (EFA) was done to assess the factor structure of the HCWCIOS items. According to the results presented in Table 1, the Kaiser–Meyer–Olkin (KMO) test value was 0.872, and Bartlett’s test value was 7468.038 (*p* < 0.001). Six factors were extracted and categorized as “Inadequate Preparedness” (eight items); “Lack of Knowledge” (seven items); “Risk Perception” (six items); “Affected Social Relations” (six items); “Work Pressure” (six items); and “Absenteeism” (three items). These six factors had eigenvalues of 9.800, 4.262, 3.051, 2.209, 1.985, and 1.797, respectively, and 46.507% of the total variance of variables of the HCWs’ concerns in the scale of the infectious outbreaks explained (Table 2). The Varimax rotation was done based on the scree plot (Figure 1) and the total variance table. Due to commonalities below 0.4, seven items were excluded from the EFA.

**Table 1:** Kaiser–Meyer–Olkin (KMO) and Bartlett’s tests for sample adequacy of HCWCIOS.

Kaiser-Meyer-Olkin measure of sampling adequacy		0.872
Bartlett’s test of sphericity	Approx. Chi-Square	7468.038
	df	1,081
	Sig.	0.000

**Table 2:** Exploratory factor analysis of HCWCIOS.

Factors	Items	Factor loading	Eigen value	Variance (%)
1. Inadequate preparedness	Q40: I feel that my organization cannot manage these patients.	0.853	9.800	10.792
	Q38: Protocols and guidelines are not fully implemented.	0.694		
	Q32: My workplace does not have a detailed plan to face the crisis caused by this pandemic.	0.664		
	Q37: The protective and preventive measures implemented in my work environment are ineffective in preventing the spread of this disease.	0.639		
	Q41: I feel that there is not enough program in our region to deal with this disease.	0.626		
	Q39: My colleagues have not taken the recommended prevention and control of infection seriously.	0.569		
	Q35: I have not received enough training on infection control and how to use personal protective equipment.	0.532		
	Q24: The rules regarding the epidemic of this disease have confused me.	0.481		
2. Lack of knowledge	Q46: I do not have enough knowledge about patient care.	0.804	4.262	9.712
	Q44: I do not have enough knowledge about the prognosis and mortality rate of this disease.	0.705		
	Q43: I do not know the signs and symptoms of this disease well enough.	0.700		
	Q47: I do not know enough to prevent and care for myself against this disease.	0.661		
	Q42: I do not know enough about this disease’s causative agent, such as its nature and ways of transmission.	0.643		
	Q45: I do not know enough about the drug treatment of this disease.	0.574		
	Q36: Most of the time, there is no one to answer my questions about this disease.	0.486		
3. Risk perception	Q3: I feel anxious while interacting with infected people.	0.853	3.051	8.220
	Q4: When communicating with infected people, the fear of transmitting the disease worries me.	0.853		
	Q6: If one of my colleagues gets this disease, I feel threatened.	0.709		
	Q14: It worries me that I do not know when the diseasewill subside.	0.525		
	Q7: I feel that I have to reduce my social activities due to the spread of this disease.	0.467		
	Q12: I am worried about the unintentional transmission of the disease to my family, friends, and colleagues.	0.415		
4. Affected social relations	Q17: I think others may stay away from my family because of my job and the possibility of getting sick.	0.733	2.209	6.622
	Q15: I think others avoid me because of my job.	0.685		
	Q19: The fear of being a disease carrier has made me stay away from my family and friends.	0.580		
	Q16: I feel that my family avoids me because I work in the hospital.	0.569		
	Q18: I am afraid to inform my family about the level of risk I am facing of being infected.	0.461		
	Q30: It is challenging for me to meet physiological needs (eating, drinking, hygiene, rest, etc.) while working.	0.402		
5. Work pressure	Q25: There are not enough human resources to carry out the affairs and demands in this situation.	0.648	1.985	6.131
	Q27: My workload has increased.	0.508		
	Q26: There are more conflicts between my colleagues and me in the work environment.	0.502		
	Q23: I feel that the organization I work for will not pay attention to my needs if I get sick.	0.499		
	Q28: Against my will, I have to work overtime.	0.498		
	Q21: I am worried that my manager and colleagues will not treat me properly if I get infected.	0.402		
6. Absenteeism	Q8: I think it is better for me to be absent from work in order not to get sick.	0.604	1.797	5.031
	Q31: I have not accepted that facing all kinds of diseases is part of the nature of my profession.	0.586		
	Q9: I feel I have to change my job because of the spread of this disease.	0.585		

The internal consistency based on Cronbach’s alpha coefficient for the inadequate preparedness factor was *α* = 0.854; for the lack of knowledge factor was *α* = 0.858; for the risk perception factor was *α* = 0.864; for affected social relations factor was *α* = 0.817; for work pressure factor was *α* = 0.754; for absenteeism was α = 0.735, and for the whole scale was *α* = 0.912. On the other hand, ICC was found as 0.880 (95% confidence interval: 0.854–0.901) by the test–retest method (Table 3).

**Table 3:** Reliability coefficient and internal consistency of HCWCIOS.

Factor	No. of item	Cronbach’s alpha coefficient	ICC	CI, 95%
				Lower – Upper
1	8	0.854	0.851	0.878–0.881
2	7	0.858	0.860	0.830–0.885
3	6	0.864	0.828	0.795–0.857
4	6	0.817	0.723	0.624–0.804
5	6	0.754	0.705	0.618–0.771
6	3	0.735	0.709	0.625–0.773
Total	36	0.912	0.880	0.854–0.901

## 4. Discussion

This study aimed to design and psychometrically evaluate a scale to measure HCWs’ concerns in infectious outbreaks. The initial scale was developed based on data obtained from extensive reviews of existing literature on HCWs’ concerns in infectious outbreaks and a qualitative study. The findings of this study confirmed that the validity and reliability of the final HCWCIOS were as expected. HCWCIOS featured 36 items and six factors: inadequate preparedness, lack of knowledge, risk perception, affected social relations, work pressure, and absenteeism.

The first factor of HCWCIOS was inadequate preparedness. Emergency preparedness involves a broad range of skills, abilities, and knowledge to prepare for and respond to catastrophes, threats, and pandemics. Understanding the readiness and preparation of HCWs to handle emergencies like the COVID-19 pandemic and deliver safe and effective treatment during these times is lacking (Chua et al., 2021). In a qualitative study, Borek et al. (2022) with the issue of HCWs’ concerns in the COVID-19 pandemic, stated that HCWs experienced substantial stress and anxiety due to the pandemic’s inadequate preparation, which was followed by requests for reflection and learning from the experience.

The second factor of this scale was the lack of knowledge. HCWs might need more knowledge regarding pandemics. As a result, individuals could not fully comprehend the risk or danger involved, which could affect their ability to stop the virus’s spread. Related studies conclude that less experienced HCWs are less knowledgeable, have lower levels of self-control and resilience, and experience greater levels of stress than more experienced HCWs who also have greater expertise (Chigwedere et al., 2021; Jamebozorgi et al., 2021). According to Malekshahi Beiranvand and Hatami Varzaneh (2018) the HCWs faced difficulties during COVID-19, including a lack of specialized expertise, inadequate readiness, and access to practical skills for managing and controlling the disease. One of the stressors identified among HCWs during the COVID-19 pandemic was a lack of knowledge and experience (Yusefi et al., 2022).

Risk perception was the third factor on the scale, with six items. Risk perception is essential in making the proper decisions during a pandemic crisis and can be viewed as the driving force behind preventive behaviors (Cori et al., 2020). According to the findings of the research done in the United States, because it was recognized that COVID-19 could result in severe effects other than death, such as serious infections and self-quarantine, the association between risk perception of COVID-19 and death due to COVID-19 has a stronger relationship with protective activities (Bruine De Bruin and Bennett, 2020). However, a cross-sectional study in Asian and European regions found no connection between awareness of the influenza pandemic risk and taking protective behaviors during the outbreak (Sadique et al., 2007). Studies showed that considering a pandemic’s perceived risk and setting standards for assessing performance can be beneficial for preventive planning, and appropriate educational interventions could be implemented (Molavi-Taleghani et al., 2020; Arefi et al., 2022).

Another factor of the designed tool was affected social relations. Several incidents of stigmatization of HCWs have emerged throughout this pandemic worldwide. In Mexico, for example, it was discovered that doctors and nurses utilize bicycles because they were allegedly denied access to public transportation and were the targets of physical assaults (Bagcchi, 2020). Healthcare professionals’ social relations studies demonstrated that HCWs’ families are psychologically impacted due to the pandemic (Lau et al., 2005; Amakiri et al., 2020). HCWs endure social stigmatization despite being praised by the media as heroes and suffer extreme anxiety and concern for their safety and the well-being of their family, friends, and coworkers. Although HCWs are more prone to seek peer psychological assistance, they also gain from being aware of the availability of official psychological support (Duffy et al., 2022). To tackle the COVID-19-related social stigma, the World Health Organization (WHO) emphasizes fostering a culture that encourages honest communication between individuals and HCWs (Bagcchi, 2020).

Work pressure was another factor. Pandemics placed extreme demands on HCWs. When pressure is high, they have had to manage a more significant number of patients with high mortality rates. They have had difficulties providing care while adhering to strict infection control procedures and not always wearing enough personal protective equipment. As a result of their redeployment into new positions, teams, or wards, many have been operating in unfamiliar settings without the established social support of their peers (Billings et al., 2021). Some studies have depicted that the unique demands of world crises and high-stress levels placed HCWs at additional risk for mental health problems (Lai et al., 2020; Greene et al., 2021).

The last but not most minor factor of HCWCIOS was absenteeism. During the SARs outbreak, several reports revealed HCWs in Toronto and Hong Kong either shied away from physical examinations of ill patients or refused to work with them because the risk they posed was too significant. At the height of China’s SARS outbreak, at least one hospital struggled to sustain services due to absenteeism, among which some were driven by concerns about getting sick (Shiao et al., 2007). More than 80% of HCWs in New York City were willing and/or able to report to work during a mass casualty or environmental disaster. However, only 57–68% would be willing to do so during a SARS or smallpox outbreak, according to a recent survey assessing their readiness for duty during a catastrophic disaster (Qureshi et al., 2005). Fears for one’s safety and the responsibilities of the family are frequently the leading causes of potential absenteeism during a pandemic, and the absenteeism rate doubles when a family member is infected (Seale et al., 2009).

The present research used a robust methodological and statistical approach to provide a valid tool for HCWs’ concerns in infectious outbreaks.

### 4.1. Limitations and strengths

The specific design to assess the HCWs’ concerns in infectious outbreaks was one of the strengths of this study. Moreover, an acceptable population diversity was recruited from different cities in Iran for the psychometric evaluation of the tool.

The most significant limitation of the current study was that access to participants was limited due to the spread of COVID-19. Furthermore, the present study was only conducted in Iran, and it is preferable to include other countries and cultures to demonstrate its trustworthiness because cultural factors can influence HCWs’ concerns. More studies are recommended to investigate this scale’s conceptual structure and to gather more evidence regarding the tool study’s psychometric properties.

## 5. Conclusion

A 36-item HCWCIOS has good psychometric properties and is suitable for measuring HCWs’ concerns during a pandemic.

## Data availability statement

The datasets presented in this study can be found in online repositories. The names of the repository/repositories and accession number(s) can be found in the article/Supplementary material.

## Ethics statement

The studies involving human participants were reviewed and approved by the Ethics Committee of Lorestan University of Medical Sciences, Khorramabad, Iran (IR.LUMS.REC.1399.007). The patients/participants provided their written informed consent to participate in this study.

## Author contributions

SY: conceptualization, methodology, data curation, writing – original draft preparation, review and editing, supervision, and project administration. MK: conceptualization, methodology, writing – original draft preparation, and review and editing. FE: formal analysis and methodology. TC: data curation and methodology. ES: formal analysis, writing – original draft preparation, review and editing. All authors contributed to the article and approved the submitted version.

## Conflict of interest

The authors declare that the research was conducted in the absence of any commercial or financial relationships that could be construed as a potential conflict of interest.

## Publisher’s note

All claims expressed in this article are solely those of the authors and do not necessarily represent those of their affiliated organizations, or those of the publisher, the editors and the reviewers. Any product that may be evaluated in this article, or claim that may be made by its manufacturer, is not guaranteed or endorsed by the publisher.
