# Supplementary material for: Development and psychometric properties of health care workers’ concerns in infectious outbreaks scale
Source: Front Psychol. 2023 Jan 17;13:1108835. doi: 10.3389/fpsyg.2022.1108835 (PMC9891178; doi:10.3389/fpsyg.2022.1108835)
Supplement: Supplementary file 1 [file Table_1.DOCX]

Supplementary Material

Development and psychometric properties of healthcare workers' concerns in infectious outbreaks scale (HCWCIOS)

**Sajad Yarahmadi^1^, Mojgan Khademi^1^, Farzad Ebrahimzadeh^2^, Tayebeh Cheraghian^1^, Elham Shahidi Delshad^3*^**

**^1^** Social Determinant of Health Research Center, School of Nursing & Midwifery, Lorestan University of Medical Sciences, Khorramabad, Iran

**^2^** Nutritional Health Research Center, School of Health and Nutrition, Lorestan University of Medical Sciences, Khorramabad, Iran

^3^ Nursing Care Research Center, Semnan University of Medical Sciences, Semnan, Iran

* Corresponding author: Email: [delshad1364@gmail.com](mailto:delshad1364@gmail.com)

**Supplementary table 1.** A developed and psychometric tool

| **No.** | **Items** | **Completely disagree** | **Disagree** | **Somewhat disagree** | **Somewhat agree** | **Agree** | **Completely agree** |
| --- | --- | --- | --- | --- | --- | --- | --- |
| 1 | I feel that my organization cannot manage these patients. |  |  |  |  |  |  |
| 2 | Protocols and guidelines are not fully implemented. |  |  |  |  |  |  |
| 3 | My workplace does not have a detailed plan to face the crisis caused by this pandemic. |  |  |  |  |  |  |
| 4 | The protective and preventive measures implemented in my work environment are ineffective in preventing the spread of this disease. |  |  |  |  |  |  |
| 5 | I feel there is not enough program in our region to deal with this disease. |  |  |  |  |  |  |
| 6 | My colleagues have not taken the recommended prevention and control of infection seriously. |  |  |  |  |  |  |
| 7 | I have not received enough training on infection control and how to use personal protective equipment. |  |  |  |  |  |  |
| 8 | The rules regarding the epidemic of this disease have confused me. |  |  |  |  |  |  |
| 9 | I don't have enough knowledge about patient care. |  |  |  |  |  |  |
| 10 | I don't know enough about this disease's prognosis and mortality rate. |  |  |  |  |  |  |
| 11 | I do not know the signs and symptoms of this disease well enough. |  |  |  |  |  |  |
| 12 | I do not know enough to prevent and care for myself against this disease. |  |  |  |  |  |  |
| 13 | I don't know enough about this disease's causative agent, such as its nature and ways of transmission. |  |  |  |  |  |  |
| 14 | I don't know enough about the drug treatment of this disease. |  |  |  |  |  |  |
| 15 | Most of the time, no one answers my questions about this disease. |  |  |  |  |  |  |
| 16 | I feel anxious while interacting with infected people. |  |  |  |  |  |  |
| 17 | The fear of transmitting the disease worries me when communicating with infected people. |  |  |  |  |  |  |
| 18 | If one of my colleagues gets this disease, I feel threatened. |  |  |  |  |  |  |
| 19 | It worries me that I don't know when the disease will subside. |  |  |  |  |  |  |
| 20 | I feel that I have to reduce my social activities due to the spread of this disease. |  |  |  |  |  |  |
| 21 | I am worried about the unintentional transmission of the disease to my family, friends, and colleagues. |  |  |  |  |  |  |
| 22 | I think that others may stay away from my family because of my job and the possibility of getting sick. |  |  |  |  |  |  |
| 23 | I think others avoid me because of my job. |  |  |  |  |  |  |
| 24 | The fear of being a disease carrier has made me stay away from my family and friends. |  |  |  |  |  |  |
| 25 | I feel that my family avoids me because I work in the hospital. |  |  |  |  |  |  |
| 26 | I am afraid to inform my family about the level of risk I am facing of being infected. |  |  |  |  |  |  |
| 27 | It is challenging for me to meet physiological needs (eating, drinking, hygiene, rest, etc.) while working. |  |  |  |  |  |  |
| 28 | There are not enough human resources to carry out the affairs and demands in this situation. |  |  |  |  |  |  |
| 29 | My workload has increased. |  |  |  |  |  |  |
| 30 | There are more conflicts between my colleagues and me in the work environment. |  |  |  |  |  |  |
| 31 | I feel that the organization I work for will not pay attention to my needs if I get sick. |  |  |  |  |  |  |
| 32 | Against my will, I have to work overtime. |  |  |  |  |  |  |
| 33 | I am worried that my manager and colleagues will not treat me properly if I get infected. |  |  |  |  |  |  |
| 34 | I think it is better for me to be absent from work to avoid getting sick. |  |  |  |  |  |  |
| 35 | I have not accepted that facing all kinds of diseases is part of the nature of my profession. |  |  |  |  |  |  |
| 36 | I feel that I have to change my job because of the spread of this disease. |  |  |  |  |  |  |
